# Call me Dr Ishmael: trends in electronic health record notes available at emergency department visits and admissions

**DOI:** 10.1093/jamiaopen/ooae039

**Published:** 2024-05-22

**Authors:** Brian W Patterson, Daniel J Hekman, Frank J Liao, Azita G Hamedani, Manish N Shah, Majid Afshar

**Affiliations:** BerbeeWalsh Department of Emergency Medicine, School of Medicine and Public Health, University of Wisconsin-Madison, Madison, WI 53705, United States; Department of Information Services, UW Health, Madison, WI 53705, United States; Department of Industrial and Systems Engineering, University of Wisconsin-Madison, Madison, WI 53705, United States; BerbeeWalsh Department of Emergency Medicine, School of Medicine and Public Health, University of Wisconsin-Madison, Madison, WI 53705, United States; BerbeeWalsh Department of Emergency Medicine, School of Medicine and Public Health, University of Wisconsin-Madison, Madison, WI 53705, United States; Department of Information Services, UW Health, Madison, WI 53705, United States; BerbeeWalsh Department of Emergency Medicine, School of Medicine and Public Health, University of Wisconsin-Madison, Madison, WI 53705, United States; BerbeeWalsh Department of Emergency Medicine, School of Medicine and Public Health, University of Wisconsin-Madison, Madison, WI 53705, United States; Department of Medicine, University of Wisconsin-Madison, Madison, WI 53705, United States; Department of Population Health Sciences, University of Wisconsin-Madison, Madison, WI 53705, United States; Department of Medicine, University of Wisconsin-Madison, Madison, WI 53705, United States

**Keywords:** summarization, documentation, natural language processing, emergency medicine

## Abstract

**Objectives:**

Numerous studies have identified information overload as a key issue for electronic health records (EHRs). This study describes the amount of text data across all notes available to emergency physicians in the EHR, trended over the time since EHR establishment.

**Materials and Methods:**

We conducted a retrospective analysis of EHR data from a large healthcare system, examining the number of notes and a corresponding number of total words and total tokens across all notes available to physicians during patient encounters in the emergency department (ED). We assessed the change in these metrics over a 17-year period between 2006 and 2023.

**Results:**

The study cohort included 730 968 ED visits made by 293 559 unique patients and a total note count of 132 574 964. The median note count for all encounters in 2006 was 5 (IQR 1-16), accounting for 1735 (IQR 447-5521) words. By the last full year of the study period, 2022, the median number of notes had grown to 359 (IQR 84-943), representing 58 662 (IQR 12 615-162 775) words. Note and word counts were higher for admitted patients.

**Discussion:**

The volume of notes available for review by providers has increased by over 30-fold in the 17 years since the implementation of the EHR at a large health system. The task of reviewing these notes has become commensurately more difficult. These data point to the critical need for new strategies and tools for filtering, synthesizing, and summarizing information to achieve the promise of the medical record.

## Background and significance

Since the early 2000s, electronic health record systems (EHRs) have been nearly universally adopted in U.S. hospital systems.^1^^,^^2^ Widespread adoption of EHRs offers an unprecedented opportunity to improve care by collecting and storing clinical data in a format instantly accessible to clinicians.^3^^,^^4^ The EHR stores information from past visits, including notes, and makes this information instantly available to treating providers to inform care.^3–5^ This information is of critical importance to providers in the emergency department (ED) as they are often meeting patients for the first time and need to provide care for acute complications of complex, chronic medical conditions. Access to prior records allows providers to understand patients’ long term and recent health history, which can be difficult to obtain from patients themselves due to health literacy, chronic conditions such as dementia, or acute conditions such as delirium that prevent effective communication.

Often a provider’s first step prior to seeing a patient in the ED is performing a “chart biopsy,” in which they briefly look through a patient’s prior notes and other data to determine pertinent medical history which could impact the present visit.^6^ Given the clinical demands on providers in the ED setting, this task is afforded a few minutes at most. However, as EHRs have grown in the scope of patient encounters documented, as well as time since deployment, the ability of any given provider to process these data effectively has become a concern in time-pressured settings such as the ED.^7–9^ In their brief patient encounters, providers in the ED are confronted with huge volumes of information, which is difficult to synthesize and act upon at the bedside.^10^^,^^11^ The expectation for a provider to review available clinical data for a given patient has remained despite an increase in the amount of data available. “Missing” critical details of a patient’s medical history—including prior diagnoses, current medications, and/or recent lab values and imaging studies—is a significant concern. These concerns exist not only for emergency providers, but also for other providers tasked with acutely caring for patients they have no previous relationship with, including hospitalists and intensivists. While prior work has suggested that the length of individual notes has increased,^12^^,^^13^ the overall quantity of text data available to clinicians in aggregate at a given clinical visit has not been evaluated.

## Objective

We sought to quantify the number and length of notes available to providers caring for patients in the ED, as measured by number of notes, total number of words among all notes, and total number of text tokens for use in a large language model (LLM). Additionally, we evaluated the trend in the amount of data presented to providers over time since the inception of the EHR system. We hypothesized that the total number of notes as well as overall text data presented to clinicians has substantially increased. As subgroup analyses, we additionally examined patients who were admitted from the ED to general care beds and intensive care units (ICUs), with the hypothesis that these patients would be more complex and have more notes.

## Methods

### Patient setting and data environment

We conducted a retrospective analysis of EHR data from quaternary care academic medical systems EHR. This EHR is used by an academic medical center that is also certified as a Level 1 Trauma. The same instance of the EHR is shared at an affiliate ED staffed by the same providers which opened in 2015, which was also included in this study. Combined, these 2 EDs now care for approximately 90 000 visits yearly, with many patients being seen at both sites. This study was IRB reviewed and granted an exemption as secondary research on existing data. The study cohort included patients aged 18 years and older at the time of an ED visit who presented to the ED between March 10, 2006 (the first year in which the EHR was deployed) and January 31, 2023. Note data were extracted from an EHR relational database (Epic Systems, Verona, WI). We included all notes available to providers (defined as physicians or advanced practice providers) during an ED encounter. This includes all information entered into the EHR as a note prior to an ED encounter, including provider-generated notes (progress notes, history and physicals, discharge summaries, and consult notes), telephone notes, and nursing and other care team notes. These notes did not include text from lab, radiology, or other procedure reports which are filed as procedures or results and not included in the “notes” section of the EHR. All notes available up to the timestamp of the index ED encounter were included, but notes generated during the encounter were not. Only notes generated within the system were included (notes from other healthcare organizations may have been available for clinician review depending on the year, but these were not analyzed for this study). Notes and encounters deemed sensitive by our institution’s policy (eg, those for patients who explicitly opted out of having their charts available for research) were excluded.

### Analysis

The unit of analysis was the patient chart as presented to the provider at the time of arrival to the ED. Data were collected at the encounter level such that 1 patient who had multiple ED encounters over the study period generated multiple data points, with the notes available at each encounter analyzed separately at each encounter. For instance, a patient may have had an encounter in 2015 where 10 notes were available, but another encounter in 2020 by which point 20 notes were available including the 10 available in 2015. This patient would result in 2 encounter data points included in the study, 1 with 10 and 1 with 20 notes available. This same patient may have been seen in 2010 while still a minor, but that encounter would be excluded from this study as an analysis point, however the notes generated during the 2010 encounter would be included as data for the 2015 and 2020 encounters.

Within each chart for each encounter, notes were individually parsed and then aggregated statistics were created by adding word and token counts from all available notes filed in the EHR prior to each ED arrival. Word count was generated by splitting text into spaces in Python (Python Software Foundation, 2019). We also generated token counts using a subword-based tokenizer (tiktoken^14^) known as byte pair encoding. This tokenizer approach is used for LLMs like GPT for tokenization and is the approach for determining the token limits for LLMs. Given a logarithmic scale was used to display data, charts which were totally empty (ie, no prior notes existed at the time of ED arrival as a patient was new to the system) had a word count of 1 assigned when displayed graphically.

Three metrics were calculated and trended by year over the study period: total number of notes, total number of words as unigrams, and total number of tokens. Median and interquartile ranges were created to quantify and compare the distribution over time. Results were graphically plotted using box plots, on a log scale to allow improved visualization of distributions. As a reference, we provided word counts of well-known English-language works to provide a benchmark for word counts. We additionally reported the patient demographics and characteristics as well as their associated note characteristics. All notes were processed in Python^15^ and the data were analyzed in R (R Development Core Team).^16^ Demographic and other clinically relevant metrics for the patients presenting were abstracted and presented for informational purposes, however, this study was not intended to examine the relationship between patient characteristics and chart size.

## Results

**Figure 1.**
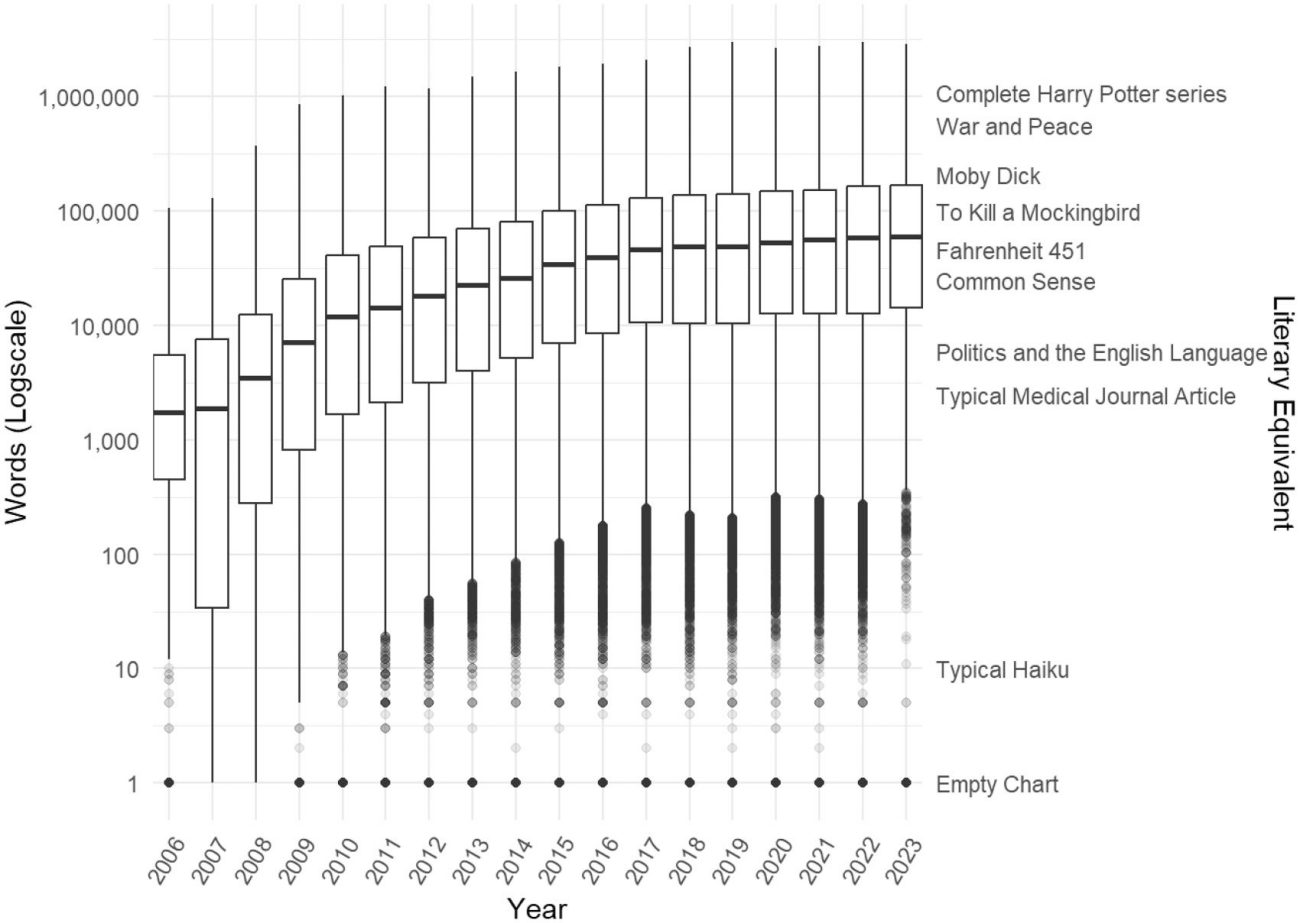
Total word count within notes available at ED visits, by year*. *Empty charts (0 words) were arbitrarily reassigned to 1 word to allow logarithmic scale display.

The study cohort included 730 968 ED visits made by 293 559 unique patients and a total note count of 132 574 964 over the 17-year study period. Figure 1 shows trends by year in the amount of note information available to providers over time by word count. Table 1 provides note counts, word length, and token count for select years, with Supplementary Appendix Table SA1 providing data for all years. The median note count for all encounters in 2006 was 5, with an interquartile range (IQR) of 1-16, accounting for 1735 words (IQR 447-5521) and 2938 tokens (IQR 784-9387). By the last full year of the study period, 2022, the median number of notes grew to 359 (IQR 84-943) notes, representing 58 662 words (IQR 12 615-162 775) and 98 308 tokens (IQR 20 819-279 576).

**Table 1.** Notes, words, and tokens available at ED encounters.

Year	ED encounters	Median (IQR) previous notes	Median (IQR) previous words	Median (IQR) previous tokens
**All encounters; all note authors**				
2010	29 220	91 (14-271)	12 011 (1650-40 747)	20 412 (2784-70 508)
2014	33 066	176 (36-512)	25 592 (5169-79 726)	43 020 (8593-137 845)
2018	59 719	290 (66-780)	48 355 (10 406-136 250)	81 000 (17 142-233 384)
2022	71 344	359 (84-943)	58 662 (12 615-162 775)	98 308 (20 819-279 576)
**ED encounters resulting in general care admission**				
2010	5568	182 (50-422)	29 288 (6670-72 070)	50 986 (11 396-128 649)
2014	4643	328 (89-794)	54 153 (13 595-136 363)	93 972 (22 988-241 014)
2018	13 103	449 (125-1061)	81 139 (21 618-198 322)	140 377 (36 428-347 990)
2022	14 383	541 (163-1278)	95 411 (24 986-235 513)	165 193 (41 844-413 185)
**ED encounters resulting in critical care (IMC/ICU) admission**				
2010	726	142 (12-390)	20 992 (2022-70 693)	38 220 (3467-127 261)
2014	754	266 (32-772)	46 936 (4464-140 960)	79 824 (7615-249 625)
2018	1879	363 (41-1017)	65 455 (6106-189 482)	113 694 (10 232-336 841)
2022	2489	424 (60-1190)	75 654 (7928-231 032)	131 720 (12 898-407 099)

Abbreviations: ED: emergency department; ICU: intensive care unit; IMC: intermediate care unit; IQR: interquartile range.

Admission data were only available on the encounter level in 2009 and after. Note, word, and token counts were larger for ED patients admitted to general care units, who by 2022 had a median of 541 notes (IQR 163-1278), accounting for 95 411 words (IQR 24 986-235 513), and patients admitted to the ICU, who in 2022 had a median of 424 notes (IQR 60-1190), accounting for 75 654 words (IQR 7928-231 032).

Tables 2 and 3 evaluate the percentiles for aggregate word counts compared to well-known literary works for notes available in 2022-January 2023, the most recent thirteen months of data collection, and those available in encounters prior to 2010 (respectively). Of note, in both Tables 2 and 3, a fraction of encounters do not exceed 0 words. These encounters represent patients who have no prior records in our health system at the time of ED check-in; this fraction has decreased from 17.27% prior to 2010 to 6.42% in 2022-2023. Descriptive statistics for patient demographics and clinical characteristics with the corresponding note length are shown in Table 4.

**Table 2.** Available note text vs literary works, January 2022 through January 2023.

Literary work	Author	Word count	Percentage of ED encounters in which available notes exceed the word count
Empty chart		0	93.58%
Typical medical journal article		2500	86.06%
*Politics and the English Language*	Orwell	5980	81.36%
*Common Sense*	Paine	25 033	66.21%
*Fahrenheit 451*	Bradbury	46 118	54.84%
*To Kill a Mockingbird*	Lee	100 388	36.34%
*Moby Dick*	Melville	206 052	19.12%
*War and Peace*	Tolstoy	561 304	3.74%
Complete* Harry Potter* series	Rowling	1 084 170	0.68%

**Table 3.** Available note text versus literary works, 2010 and prior.

Literary work	Author	Word count	Percentage of ED encounters in which available notes exceed the word count
Empty chart		0	82.73%
Typical medical journal article		2500	56.70%
*Politics and the English Language*	Orwell	5980	42.59%
*Common Sense*	Paine	25 033	17.13%
*Fahrenheit 451*	Bradbury	46 118	9.18%
*To Kill a Mockingbird*	Lee	100 388	2.96%
*Moby Dick*	Melville	206 052	0.63%
*War and Peace*	Tolstoy	561 304	0.03%
Complete* Harry Potter* series	Rowling	1 084 170	0.00%

**Table 4.** Demographics over entire study period.

	*n*	Previous note count, median (IQR)	Previous note words, median (IQR)	Previous note tokens, median (IQR)
Overall	730 968	184 (28-597)	28 611 (4274- 98 967)	48 115 (7121-169 947)
Age group				
18-34	222 810	53 (5-239)	7926 (636-36 823)	13 071 (1063-60 934)
35-64	334 610	206 (44-602)	31 858 (6599-98 526)	53 690 (11 054-168 965)
65+	173 548	476 (145-1026)	80 120 (23 776-181 844)	138 972 (40 895-317 737)
Patient biologic sex				
Female	394 083	234 (43-684)	35 157 (6376-110 772)	59 274 (10 668-189 662)
Male	336 869	134 (17-492)	21 802 (2591-85 009)	36 581 (4302-146 102)
Non-binary or other	16	12 (5-136)	1996 (737-21 268)	2942 (1139-34 286)
Patient race (first listed)				
American Indian or Alaska native	8238	115 (15-409)	17 950 (2318-67 516)	30 150 (3910-114 887)
Asian	21 524	39 (2-230)	5942 (99-37 394)	9824 (165-62 469)
Black or African American	83 007	184 (33-606)	28 371 (5000-99 598)	47 569 (8308-169 955)
Native Hawaiian or other Pacific Islander	1356	97 (12-344)	17 308 (1504-57 232)	28 670 (2513-95 662)
White	607 669	196 (31-619)	30 542 (4783-102 882)	51 457 (7981-176 833)
Not recorded	9174	37 (2-212)	5840 (205-35 222)	9688 (349-59 114)
Patient ethnicity				
Hispanic/Latino	34 822	83 (10-311)	13 254 (1410-52 865)	22 214 (2351-89 076)
Not Hispanic or Latino	691 697	193 (30-615)	29 911 (4639-102 302)	50 357 (7729-175 573)
Not recorded	4449	14 (0-176)	2159 (0-26 929)	3543 (0-45 227)
Interpreter needed for ED encounter?				
Non-English interpreter needed	18 330	69 (7-295)	11 658 (954-54 232)	19 798 (1601-93 470)
No interpreter needed	712 638	188 (29-605)	29 194 (4442-100 208)	49 081 (7392-172 001)
Means of arrival				
Family/friend/self	532 879	164 (26-522)	25 264 (4012-85 038)	42 349 (6664-145 024)
EMS	175 767	293 (44-889)	47 141 (6958-154 242)	80 418 (11 680-268 168)
Helicopter	5094	4 (2-49)	541 (75-7635)	976 (130-13 034)
Other or not recorded	17 228	137 (11-587)	23 589 (2156-101 510)	39 675 (3532-174 280)
Emergency severity index				
Level 1: Red	5482	57 (3-399)	9308 (318-68 056)	15 500 (559-117 540)
Level 2: Pink	154 528	244 (40-723)	39 727 (6423-127 780)	67 762 (10 761-222 794)
Level 3: Yellow	446 633	205 (36-628)	31 672 (5551-103 728)	53 395 (9263-178 037)
Level 4: Green	113 327	82 (8-345)	12 288 (1238-52 906)	20 426 (2058-88 596)
Level 5: Blue	9724	65 (7-311)	9926 (986-48 316)	16 361 (1625-80 275)
Not recorded	1274	77 (4-426)	12 607 (1097-69 981)	20 950 (1812-116 116)
ED disposition				
Discharge	547 476	154 (23-511)	23 365 (3439-81 554)	39 073 (5719-138 592)
Admit	172 031	311 (52-862)	52 924 (8972-156 670)	91 271 (15 220-275 204)
Transfer	11 461	394 (92-1030)	65 938 (14 516-177 407)	111 899 (24 237-308 678)
Has in-system primary care provider?				
In-system PCP	412 830	352 (111-856)	55 642 (16 952-145 491)	93 896 (28 315-250 330)
PCP not affiliated with ED system or No PCP	318 138	44 (3-245)	6 724 (269-39 433)	11 247 (462-67 336)

Abbreviations: ED: emergency department; IQR: Interquartile range.

## Discussion

Numerous prior studies have examined the general state of information overload in the EHR and its contribution to burnout.^7–9^ Other work has specifically examined the phenomenon of note bloat, in which over time EHR notes have become longer and more repetitive,^12^^,^^17^ leading to stress among providers responsible for reading and creating notes.^18^^,^^19^ Less attention, however, has been paid to the growth of the overall chart, and the related burden at the bedside for providers who routinely care for patients with whom they do not have an established prior relationship. Our findings demonstrate that the number of notes available to physicians in the EHR has significantly increased over the past decade, adding quantifiable evidence to the discussion on EHR-related physician burden. This highlights the increasing difficulty of an unassisted human “chart biopsy” task, especially in the ED. Emergency physicians often are responsible for caring for 3 or more new patients per hour,^20^ giving them an average time of 20 minutes for all patient care tasks: chart biopsy, face-to-face interview of the patient, physical examination, ordering and interpretation of test results, administration of therapies and procedures, discussions with consultants, writing discharge instructions, and counseling patients. Only a small fraction of these 20 min can be safely devoted to chart biopsy without affecting other critical patient care activities. While the time available for chart review has not increased, our results suggest that the chart biopsy task has drastically grown in magnitude since the inception of the EHR.

In this study, we used well-known literary works to benchmark the volume of text data available in patients’ charts at the time of ED presentation. Prior to 2010, the task for a median patient was analogous to skimming a brief essay such as Orwell’s *Politics of the English Language* (6 000 words) to identify any potential salient points. This is difficult, but possible within a few minutes window. Today, a chart biopsy for the median patient is more analogous to skimming *Fahrenheit 451* (a 46 000-word novel), while nearly 1 in 5 patients arrive with a chart the size of *Moby Dick* (209 117 words). Skimming *Moby Dick* and identifying all possible health concerns for Captain Ahab is not a task a human can perform within the constraints of an ED visit. Inpatient and ICU providers may have more time to conduct a chart review, but their task has grown to an even larger proportion: the average admitted patient has a chart over twice as large a word count as those examined during general ED visits. The analogy between charts and literary works is imperfect as words are organized very differently in a novel than a chart; we provide these comparisons purely to provide benchmarks for the number of available words in charts.

These findings do not suggest that it is inherently wrong to store large amounts of text data within the EHR. The centralized, accessible storage of medical notes is a key benefit of EHR software, and studies have shown that even in the time-constrained ED setting, access to prior records improves physicians’ diagnostic ability.^21^ Since the time of early EHR adoption, there has been an understanding that there is a tension between the EHR’s function for documentation storage and its need to provide retrievable information to support clinical tasks.^22^ However, as the volume of textual data continues to grow, this tension has become irreconcilable without additional tools to filter and summarize information. In the current state, providers cannot read all or even a small fraction of the notes available to them during a chart biopsy and must use filters and other tools to sort information. While filters can help simplify views, these approaches risk missing critical pieces of data. The vast increase in the magnitude of text stored points to the need for more sophisticated solutions to ensure patient safety. Prior studies have suggested strategies aimed at improving clinicians’ ability to curate or retrieve EHR data at the bedside,^23^ but these have not achieved widespread implementation. The potential application of LLMs offers a promising avenue for addressing the information overload challenge in EHRs.^24^ These models can be trained to generate concise and relevant summaries of patient notes, allowing physicians to quickly grasp essential information without sifting through extensive text data. Newer approaches in retrieval augmented generation with LLMs can retrieve relevant textual data, reducing factual errors in knowledge-intensive tasks with the potential to reduce the cognitive load on the physician.^25^

One limitation of current LLMs, however, is the constraint on the number of tokens that can be processed at once. For example, GPT-4 Turbo has a token input limit of up to 128 000 tokens. Most open-source LLMs have smaller token limits. These token limits pose challenges for summarizing large numbers of notes and highlight the need for research into strategies to both filter the input into such models and improve their ability to ingest large amounts of data for summarization if they are to provide meaningful impact in improving care.

## Conclusion

This study quantifies the escalating challenge created by the volume of text data confronting providers while caring for multiple simultaneous patients in the time-pressured environments of the ED and at admission to inpatient units. While the central storage of medical notes remains a critically important function for the EHR, our findings emphasize the urgency of addressing the resultant information overload for healthcare providers. While LLMs offer a potential venue for summarization, the volume of the summarization task may provide challenges for currently available strategies to use these models efficiently. Further research and development in the field of natural language processing will be crucial to assist healthcare providers in navigating the information-rich landscape of EHRs efficiently and effectively to provide optimal patient care.

## Supplementary Material

ooae039_Supplementary_Data

## Data Availability

The data underlying this article cannot be shared publicly due to privacy concerns, as the dataset is comprised of identifiable patient notes. On reasonable request to the corresponding author, data may be shared after the completion of an IRB and execution of the appropriate data use agreement.
