# Correction to: Call me Dr Ishmael: trends in electronic health record notes available at emergency department visits and admissions

**DOI:** 10.1093/jamiaopen/ooae063

**Published:** 2024-06-26

This is a correction to: Brian W Patterson *et al.*, Call me Dr Ishmael: trends in electronic health record notes available at emergency department visits and admissions, *JAMIA Open*, Volume 7, Issue 2, July 2024, ooae039, https://doi.org/10.1093/jamiaopen/ooae039

The results section of the abstract has been updated to correct a typographical error: the phrase ‘representing 359 (IQR 84-943) words’ has been updated to read ‘representing 58 662 (IQR 12 615-162 775) words’.

