# Supplementary material for: Call me Dr Ishmael: trends in electronic health record notes available at emergency department visits and admissions
Source: JAMIA Open. 2024 May 22;7(2):ooae039. doi: 10.1093/jamiaopen/ooae039 (PMC11110617; doi:10.1093/jamiaopen/ooae039)
Supplement: ooae039_Supplementary_Data [file ooae039_supplementary_data.docx]

## Appendix 1. Notes, Words, and Tokens Over Time – All Years

| Year | ED Encounters | Median (IQR) Previous Notes | Median (IQR) Previous Words | Median (IQR) Previous Tokens |
| --- | --- | --- | --- | --- |
| **All ED Encounters** | | | | |
| 2006 | 19,507 | 5 (1 - 16) | 1,735 (447 - 5,521) | 2,938 (784 - 9,387) |
| 2007 | 27,621 | 10 (1 - 36) | 1,886 (33 - 7,555) | 3,283 (58 - 13,074) |
| 2008 | 23,106 | 23 (3 - 74) | 3,488 (278 - 12,286) | 6,014 (502 - 21,331) |
| 2009 | 23,494 | 49 (7 - 155) | 7,036 (820 - 25,115) | 11,954 (1,390 - 43,212) |
| 2010 | 29,220 | 91 (14 - 271) | 12,011 (1,650 - 40,747) | 20,412 (2,784 - 70,508) |
| 2011 | 30,076 | 116 (20 - 349) | 14,361 (2,125 - 48,549) | 24,546 (3,572 - 84,543) |
| 2012 | 31,028 | 145 (28 - 416) | 18,037 (3,157 - 57,764) | 30,709 (5,254 - 100,110) |
| 2013 | 32,152 | 165 (32 - 471) | 22,249 (4,016 - 69,041) | 37,608 (6,641 - 119,719) |
| 2014 | 33,066 | 176 (36 - 512) | 25,592 (5,169 - 79,726) | 43,020 (8,593 - 137,845) |
| 2015 | 45,142 | 222 (47 - 616) | 34,088 (6,944 - 100,365) | 57,528 (11,470 - 173,390) |
| 2016 | 52,890 | 244 (53 - 669) | 39,383 (8,538 - 112,680) | 66,133 (14,091 - 193,768) |
| 2017 | 56,657 | 275 (63 - 732) | 46,150 (10,634 - 129,163) | 77,476 (17,484 - 222,417) |
| 2018 | 59,719 | 290 (66 - 780) | 48,355 (10,406 - 136,250) | 81,000 (17,142 - 233,384) |
| 2019 | 64,093 | 296 (66 - 804) | 48,937 (10,325 - 140,338) | 82,005 (16,995 - 240,287) |
| 2020 | 57,674 | 326 (81 - 864) | 53,124 (12,712 - 149,098) | 89,311 (20,970 - 255,811) |
| 2021 | 68,119 | 342 (83 - 881) | 56,285 (12,618 - 152,589) | 94,123 (20,736 - 262,056) |
| 2022 | 71,344 | 359 (84 - 943) | 58,662 (12,615 - 162,775) | 98,308 (20,819 - 279,576) |
| 2023 | 6,060 | 366 (96 - 958) | 59,126 (14,119 - 166,747) | 99,492 (23,436 - 286,147) |
| **ED Encounters Resulting in General Care Admission** | | | | |
| 2009 | 1,594 | 132 (34 - 327) | 23,234 (5,284 - 61,325) | 40,449 (9,304 - 109,084) |
| 2010 | 5,568 | 182 (50 - 422) | 29,288 (6,670 - 72,070) | 50,986 (11,396 - 128,649) |
| 2011 | 4,916 | 240 (66 - 552) | 34,808 (8,658 - 90,577) | 61,298 (14,798 - 163,230) |
| 2012 | 5,261 | 285 (79 - 659) | 40,315 (10,196 - 104,275) | 70,585 (17,309 - 186,260) |
| 2013 | 5,760 | 304 (82 - 703) | 46,384 (11,553 - 114,160) | 80,614 (19,265 - 204,466) |
| 2014 | 4,643 | 328 (89 - 794) | 54,153 (13,595 - 136,363) | 93,972 (22,988 - 241,014) |
| 2015 | 12,637 | 358 (98 - 855) | 60,469 (15,664 - 148,842) | 104,642 (26,634 - 263,437) |
| 2016 | 13,370 | 398 (112 - 950) | 69,894 (18,723 - 172,662) | 121,084 (31,736 - 304,286) |
| 2017 | 13,153 | 436 (128 - 1,035) | 80,991 (22,622 - 194,124) | 140,220 (38,455 - 341,617) |
| 2018 | 13,103 | 449 (125 - 1,061) | 81,139 (21,618 - 198,322) | 140,377 (36,428 - 347,990) |
| 2019 | 12,341 | 469 (132 - 1,075) | 83,190 (22,497 - 199,254) | 143,686 (38,253 - 350,797) |
| 2020 | 12,101 | 494 (148 - 1,158) | 85,730 (24,830 - 214,029) | 147,706 (41,678 - 375,749) |
| 2021 | 14,227 | 537 (157 - 1,216) | 94,115 (25,428 - 226,708) | 162,794 (42,773 - 397,156) |
| 2022 | 14,383 | 541 (163 - 1,278) | 95,411 (24,986 - 235,513) | 165,193 (41,844 - 413,185) |
| 2023 | 1,166 | 603 (212 - 1,342) | 108,964 (33,729 - 246,710) | 190,650 (57,724 - 429,744) |
| **ED Encounters Resulting in IMC/ICU Admissions** | | | | |
| 2009 | 239 | 64 (2 - 250) | 10,575 (170 - 49,006) | 18,277 (276 - 85,228) |
| 2010 | 726 | 142 (12 - 390) | 20,992 (2,022 - 70,693) | 38,220 (3,467 - 127,261) |
| 2011 | 826 | 178 (10 - 544) | 27,366 (1,222 - 98,353) | 48,730 (2,030 - 183,097) |
| 2012 | 938 | 253 (16 - 641) | 36,396 (1,784 - 109,536) | 65,454 (3,065 - 199,874) |
| 2013 | 1,174 | 217 (19 - 678) | 33,986 (2,303 - 118,662) | 59,100 (3,605 - 217,762) |
| 2014 | 754 | 266 (32 - 772) | 46,936 (4,464 - 140,960) | 79,824 (7,615 - 249,625) |
| 2015 | 1,839 | 213 (14 - 696) | 37,309 (1,417 - 130,614) | 65,171 (2,527 - 229,620) |
| 2016 | 1,931 | 267 (30 - 871) | 49,127 (4,114 - 157,350) | 84,267 (6,694 - 277,600) |
| 2017 | 1,738 | 325 (37 - 929) | 57,458 (5,967 - 176,503) | 99,575 (9,722 - 309,064) |
| 2018 | 1,879 | 363 (41 - 1,017) | 65,455 (6,106 - 189,482) | 113,694 (10,232 - 336,841) |
| 2019 | 2,048 | 338 (58 - 971) | 59,097 (9,086 - 187,422) | 101,410 (15,576 - 332,367) |
| 2020 | 2,168 | 332 (45 - 948) | 60,157 (7,062 - 177,361) | 103,038 (11,625 - 308,692) |
| 2021 | 2,262 | 278 (25 - 906) | 48,904 (3,408 - 171,488) | 83,261 (5,680 - 301,309) |
| 2022 | 2,489 | 424 (60 - 1,190) | 75,654 (7,928 - 231,032) | 131,720 (12,898 - 407,099) |
| 2023 | 224 | 476 (88 - 1,095) | 98,132 (10,316 - 228,736) | 167,291 (17,159 - 411,804) |
| IMC: Intermediate Care Unit; ICU: Intensive Care Unit; IQR: Interquartile Range | | | | |
